# An Exhibit of Historic Interest

**Published:** 1904-11

**Authors:** 


### An Exhibit of Historic Interest.

A HISTORIC exhibition of rare and curious objects relating to medicine, chemistry, pharmacy and the allied sciences will be held shortly in London under the direction of Mr. Henry S. Wellcome, of the firm of Burroughs, Wellcome & Company, well-known English manufacturers of pharmaceuticals. This exhibition will be something in the nature of a celebration of the twenty-fifth anniversary of the founding of the firm, and will bring together a collection of material illustrating the development of the art and science of healing from the earliest period up to the present time, from the crudest and most primitive methods and drugs to the elegant and finished preparations of today.

For many years Mr. Wellcome has been studying early methods of healing among the civilised and uncivilised races, and from time to time he has issued booklets descriptive of his work, which are among the most interesting publications on the subject. Naturally, the success of the exhibition will depend largely upon the coöperation of those who have made even a perfunctory study of the development of medicine and their willingness to loan whatever they may have in the way of illustrations, crude drugs, early pictorial plates, amulets, relics of medical practice instruments. The expense of transportation and insurance will be borne by Mr. Wellcome, who may be communicated with at Snow Hill Building, London, E. C. An idea of the scope of the exhibition may be gathered from the following classification of exhibits, which has been prepared as a guide:

### CLASSIFICATION OF EXHIBITS.

Section 1.—Paintings, drawings, engravings, prints, photographs, silhouettes, medallions, sculptures and casts of historical interest, of: (a) distinguished physicians, surgeons, alchemists, apothecaries, chemists, pharmacists, nurses, etc.; (b) antient British and foreign medical, chemical and pharmaceutical institutions;

(c) important and interesting events in the history of medicine, chemistry, and pharmacy.

Section 2.—Rare and curious manuscripts, incunabula, books, periodicals, pamphlets and book-plates on, and connected with, medical, chemical, pharmaceutical and allied scientific subjects.

Section 3.—Letters, prescriptions, autographs, records of experiments, antient diplomas, licenses, instruments, apparatus, and other personal relics of medical, pharmaceutical and chemical interest.

Section 4.—Curiosities of medicine: (a) materia medica of all ages. Specimens of antient medicines in all forms; (b) formulæ of all ages; (c) antient and modern medicine chests—civil, military and naval; (d) votive offerings for health; antient and modern amulets, amuletic medicines, medals, tokens, seals, emblems, charms and talismans. Medical relics of savage and primitive peoples; (e) antient corporate insignia and early diplomas in medicine granted by British and foreign colleges; (f) rare and curious memorials of medical practice; (g) memorials of medication by animal substances; (h) memorials of the influence of astrology on medicine.

Section 5.—Curiosities of surgery: (a) relics of antient and mediæval surgery, dentistry and veterinary surgery; (b) antient and mediæval hospital equipment; (c) curiosities of anatomy, and curious anatomical models; (d) historical and antient surgical instruments, appliances, etc.; (e) antient corporate insignia and early diplomas in surgery granted by British and foreign colleges; (f) instruments used in surgery by primitive peoples.

Section 6.—Curiosities of pharmacy: (a) quaint pharmaceutical recipes; (b) scales, weights and measures of all ages; (c) antient stills, mortars and pharmaceutical implements; (d) curious bottles, carboys, retorts, alembics, ointment jars, drug jars, ewers, mills, etc.; (e) curious laboratory apparatus; (f) antient prescription-books and price lists; (g) antient counter bills, labels, business cards, curious advertisements and trade tokens; (h) antient apothecaries' shop-signs, early shop fittings and appliances; (i) early pharmaceutical specialties, and specimens of obsolete and strange medical combinations; (j) old travelers' advice-books, curious orders, and the like.

Section 7.—Curiosities of allied sciences: (a) antient herbaria--abnormal plant forms; (b) curious magnetic and electrical appliances; (c) curious relics of dental surgery. Early artificial dentures. Antient instruments, etc.

Section 8.—Historical apparatus associated with important discoveries in medicine, chemistry and pharmacy.

Section 9.—Preventive medicine. Public health. Tropical medicine. Objects of interest, antient and modern, connected with the treatment of plague and pestilence. Exhibits illustrative of physiology, anthropology, microscopy, bacteriology, biology and geography, toxicology (curious poisons and historical objects connected with famous poisoning cases).

Section 10.—Nursing and ambulance: Early hospital and general nursing. Infant nursing. Ambulance appliances. Antient feeding cups and bottles. Naval and military nursing and ambulance appliances and equipments. Portraits of famous nurses. Relics and objects of interest associated with nurses.

Section 11.—Curiosities of photography: Objects illustrating the invention and history of photography. Early cameras and apparatus. Daguerreotypes. Portraits of the pioneers of photography. Original papers and early MSS. on photography. Application of photography to medicine. Early *x*-ray apparatus.

Section 12.—Adulteration and falsification of drugs, medicines, foodstuffs, fabrics, and of any articles affecting health, or associated with medicine, chemistry, pharmacy and allied sciences.

